# A Two-Step Target Binding and Selectivity Support Vector Machines Approach for Virtual Screening of Dopamine Receptor Subtype-Selective Ligands

**DOI:** 10.1371/journal.pone.0039076

**Published:** 2012-06-15

**Authors:** Jingxian Zhang, Bucong Han, Xiaona Wei, Chunyan Tan, Yuzong Chen, Yuyang Jiang

**Affiliations:** 1 The Key Laboratory of Chemical Biology, Guangdong Province, Graduate School at Shenzhen, Tsinghua University, Shenzhen, People's Republic of China; 2 Bioinformatics and Drug Design Group, Department of Pharmacy, Centre for Computational Science and Engineering, National University of Singapore, Singapore, Singapore; 3 Computation and Systems Biology, Singapore-MIT Alliance, National University of Singapore, Singapore, Singapore; University of Helsinki, Finland

## Abstract

Target selective drugs, such as dopamine receptor (DR) subtype selective ligands, are developed for enhanced therapeutics and reduced side effects. *In silico* methods have been explored for searching DR selective ligands, but encountered difficulties associated with high subtype similarity and ligand structural diversity. Machine learning methods have shown promising potential in searching target selective compounds. Their target selective capability can be further enhanced. In this work, we introduced a new two-step support vector machines target-binding and selectivity screening method for searching DR subtype-selective ligands, which was tested together with three previously-used machine learning methods for searching D1, D2, D3 and D4 selective ligands. It correctly identified 50.6%–88.0% of the 21–408 subtype selective and 71.7%–81.0% of the 39–147 multi-subtype ligands. Its subtype selective ligand identification rates are significantly better than, and its multi-subtype ligand identification rates are comparable to the best rates of the previously used methods. Our method produced low false-hit rates in screening 13.56 M PubChem, 168,016 MDDR and 657,736 ChEMBLdb compounds. Molecular features important for subtype selectivity were extracted by using the recursive feature elimination feature selection method. These features are consistent with literature-reported features. Our method showed similar performance in searching estrogen receptor subtype selective ligands. Our study demonstrated the usefulness of the two-step target binding and selectivity screening method in searching subtype selective ligands from large compound libraries.

## Introduction

Drugs that selectively modulate protein subtypes are highly useful for achieving therapeutic efficacies at reduced side effects [1], [2], [3], [4]. For some targets such as dopamine receptors, all of the approved drugs are subtype non-selective, and this non-selectivity directly contributes to their observed side effects and adversely affects their application potential [4]. There is a need for developing subtype selective drugs against these targets [3], [4], [5], [6], [7].

The drug-binding domains of some protein subtypes are highly similar to each other. For instance, the sequence similarities among the transmembrane regions of dopamine receptor subtypes are at high levels of 72%, 73% and 90% between D2-like subfamily members D2 and D4, D3 and D4, and D2 and D3 respectively [8], and at the levels of 68%, 70% and 66% between D1 and D2, D1 and D3 and D1 and D4 respectively. Ligand binding selectivity to these subtypes is both determined by the structural and physicochemical features of the conserved and non-conserved residues [9]. For instance, while D2 receptor and D3 receptor share high sequence identity in the seven helices regions that make up most of the binding sites, different compositions of the loop regions affect the contour and topography of the binding pockets and hydrogen bonding sites, which enables subtype selective binding [10], [11]. On the other hand, D2/D4 selectivity has been suggested to be determined by mutated residues within the second, third, and seventh membrane-spanning segments [9].

The high sequence similarity levels make it more difficult to develop dopamine receptor subtype-selective drugs. Efforts have been made in exploring *in-silico* methods for searching dopamine receptor subtype-selective drug leads against highly similar subtypes. For instance, 3D-QSAR models have been developed for D2, D3 and D4 selective ligands respectively, achieving good prediction performances with R^2^ and Q^2^ values in the ranges of 0.89–0.97 and 0.58–0.84 respectively [10], [11], [12], [13]. A GALAHAD based selective pharmacophore model has been constructed for D1/D2 selective agents [14]. CoMFA and CoMSIA models have been developed for D2, D3 and D4 selective ligands [15].

These models have been developed by using 12–163 ligands. Significantly higher numbers of dopamine receptor ligands including subtype selective [2], [4] and multi-subtype [16], [17] ligands have been reported. These ligands are of high structural diversity. The published D1, D2, D3 and D4 ligands are distributed in 225, 642, 463 and 433 compound families (**Table** **1**) compared to the 90–388 families covered by the inhibitors of many kinases [18]. These structurally diverse ligands are not expected to be fully presented by the existing models trained from limited numbers of ligands. More extensive exploration of the available ligands is needed for developing more effective *in-silico* tools for searching subtype-selective dopamine receptor ligands.

**Table 1:** Datasets of our collected dopamine receptor D1, D2, D3 and D4 ligands, non-ligands and putative non-ligands.

	Training Dataset			Independent Testing Dataset	
Dopamine Receptor Subtype	Positive Samples	Negative Samples		Positive Samples	Negative Samples
	Ligands published before 2010 (No of chemical families covered by ligands)	Non-ligands published before 2010	Putative non-ligands	Ligands published since 2010 (percent of ligands outside training chemical families)	Non-ligands published since 2010
D1	491 (225)	264	65198	59 (25.42%)	25
D2	2202 (642)	1577	63687	135 (16.30%)	65
D3	1355 (463)	631	62927	76 (18.42%)	28
D4	1486 (433)	526	63272	29 (34.48%)	33

Dopamine receptor D1, D2, D3 and D4 ligands (Ki <1 μM) and non-ligands (ki >10 μM) were collected as described in method section, and putative non-ligands were generated from representative compounds of compound families with no known ligand. These datasets were used for training and testing the multi-label machine learning models.

Machine learning methods are particularly useful for developing virtual screening (VS) models from structurally diverse compounds and for searching large chemical libraries [19], [20], [21]. The purchasable real chemical libraries have been expanded to >1 million compounds [22] and the public chemical databases have been expanded at faster paces with PubChem [23], ZINC [24], and ChEMBL [25] databases accumulating >30 million compounds, >13 million purchasable compounds, and >1 million bioactive compounds respectively. The available chemical space defined by these databases may be more extensively explored by the use of machine learning methods [26], [27].

Moreover, several multi-label machine learning methods have been used for developing *in-silico* tools to predict protein selective compounds within a protein family or subfamily. For instance, multi-label support vector machines (ML-SVM), multi-label k-nearest-neighbor (ML-kNN) and multi-label counter-propagation neural network (ML-CPNN) methods have been used for predicting isoform specificity of P450 substrates [28], [29]. Combinatorial support vector machines (Combi-SVM) method has been used for identifying dual kinase inhibitors selective against single kinase inhibitors of the same kinase pair and inhibitors of other kinases [18]. It is of interest to explore some of these methods and to evaluate their capability in predicting subtype selective dopamine receptor ligands.

These existing methods are based on statistical learning algorithms trained by compounds active and inactive against a specific protein or subtype [18], [19], [28], [29]. In these algorithms, the inactive chemical space can be represented by a large number of inactive compounds in a training dataset that typically include representative compounds of chemical families or biological classes. In particular the inactive training dataset of a subtype is typically too large to further add sufficient number of active compounds of other subtypes [18], [19], [28], [29]. Consequently, although these methods have shown good performance in selecting ligands of a subtype, they do not always distinguish subtype selective and non-selective ligands at good accuracy levels. For instance, the ML-SVM, ML-kNN and ML-CPNN methods predict 34%–89% isoform selective substrates as selective and 82%–99% isoform non-selective substrates as non-selective [28]. Combi-SVM identifies 51.9%–96.3% single kinase inhibitors as kinase selective with respect to a specific kinase pair and 12.2%–57.3% dual kinase inhibitors as dual inhibitors [18]. Therefore, new methods need to be explored for better distinguishing subtype selective and non-selective ligands.

In this work, we introduced a new method, the two-step binary relevance SVM (2SBR-SVM) method for improving the ability in distinguishing subtype selective and non-selective ligands. Our method adopts a two-step approach, with the first step focusing on the identification of putative ligands of a receptor subtype regardless of their possible binding to other subtypes, and the second step focusing on the further separation of subtype selective and multi-subtype ligands. In the first step, a SVM model was developed for each receptor subtype to select putative ligands regardless of their possible binding to other subtypes using the same method as that described in our earlier studies [19]. In the second step, the Binary relevance (BR) method [30] was used for more refined separation of subtype selective and multi-subtype ligands. Specifically, the training datasets of the multiple receptor subtypes were re-arranged into multiple new training datasets, one for each subtype. For a particular subtype, the ligands of that subtype were used as positive samples and the ligands of the other subtypes as the negative samples to train a SVM model for maximally separating ligands of a subtype with those of other subtypes.

Our new method 2SBR-SVM was tested together with three previously-used methods Combi-SVM [18] and two methods in the Mulan software package [30]: the ML-kNN [28], [31] and Random k-labelset Decision Tree (RAkEL-DT) [32], [33] methods. The purpose of these tests was to evaluate the performance of the previously used methods, and to determine to what extent our new method can improve the performance in selecting dopamine subtype selective ligands.

A number of dopamine receptor subtype selective ligands have been therapeutically explored. For instance, most currently used dopamine agonists for the symptomatic treatment of Parkinson's disease are selective for D2-like receptors primarily because drugs acting on both the D1 and D2 receptor families tend to additively produce motor complications such as dyskinesia [34]. D2-selective drugs have exhibited therapeutic efficacy in animal studies [35] and clinical trials [36]. D3-selective drugs have been explored for the treatment of schizophrenia and drug addiction [37], [38]. D4-selective ligands have shown therapeutic potential against erectile dysfunction [39], [40]. Efforts have also been directed to the development of D1-selective [41], [42] ligands against Parkinson's disease and other related CNS disorders. Therefore, our tests were conducted on D1, D2, D3 and D4 selective and non-selective ligands.

Our VS models were trained from 491–2202 dopamine receptor D1, D2, D3, and D4 ligands published before 2010 with all the known subtype selective ligands and some known multi-subtype ligands excluded. The reason for the exclusion of these subtype selective and multi-subtype ligands from the training process is to test to what extent our VS models can identify subtype selective ligands without explicit knowledge of the known subtype selective and multi-subtype ligands. The prediction performance of these models was evaluated by 29–135 known D1, D2, D3 and D4 ligands and 25–65 known non-ligands published since 2010 and not in the training datasets. The subtype selectivity of these models was tested on the 21–408 known subtype selective ligands and the 39–147 known multi-subtype ligands not in the training datasets.

The performance of our new method, 2SBR-SVM, and the method developed in our previous studies, Combi-SVM [18], in screening large compound libraries was evaluated by 13.56 million PubChem compounds [23], 168,016 MDL Drug Data Report (MDDR) database compounds, and 657,736 ChEMBLdb compounds [43] which represent general chemical libraries, patented bioactive agents, and published bioactive compounds respectively. The capability of 2SBR-SVM in identifying subtype selective ligands of other receptors was further evaluated against estrogen receptor (ER) ERα and ERβ subtype ligands by using the same training and testing procedures as those of the dopamine receptor subtype ligands.

## Methods

### Datasets

D1, D2, D3 and D4 ligands and non-ligands were collected from comprehensive search of literatures [38], [41], [44], [45] and ChEMBLdb database [43] by using combinations of keywords: “dopamine”, “D1 receptor”, “D2 receptor”, “D3 receptor”, “D4 receptor”, “ligand”, “binding”, “binder”, “subtype selective”, and “selective ligand”. As the ligands were collected from different sources with their binding affinities measured under different assays and conditions, some level of variations in binding affinities is expected. Therefore, we tentatively selected compounds with binding affinity Ki <1 μM against a dopamine receptor as its ligands, and those with binding affinity Ki >10 μM as non-ligands. The 1 μM to 10 μM binding affinity gap between ligands and non-ligands was used for reducing the possible influence of experimental binding affinity variations on the robustness of developed VS models. Some of the dopamine receptor ligands have been explicitly reported to be subtype selective or multi-subtype ligands, which can be used for testing the subtype selective capability of our developed VS models. Thus for subtypes with ≥20 subtype selective or ≥20 multi-subtype ligands, the corresponding ligands were used as independent testing datasets (a cut-off of 20 ligands was used to ensure the testing to be statistically meaningful).

We assembled 491 D1, 2202 D2, 1355 D3 and 1486 D4 ligands published before 2010 and 59 D1, 135 D2, 76 D3 and 29 D4 ligands published since 2010 with unspecified selectivity toward other subtypes, and 264 D1, 1577 D2, 631 D3 and 526 D4 non-ligands published before 2010 and 25 D1, 65 D2, 28 D3 and 33 D4 non-ligands published since 2010 with unspecified selectivity toward other subtypes. The collected pre-2010 ligands and non-ligands for each receptor subtype were used as positive and negative samples of the training dataset for developing VS models for that subtype. The collected non-ligands are insufficient to cover the vast non-ligand chemical space. Therefore, putative ligands for each receptor subtype were generated from the representative compounds of the compound families that contain no known ligand of that subtype by using the method described in our earlier studies [19]. A total of 65198 D1, 63687 D2, 62927 D3 and 63272 D4 putative non-ligands were generated and used in combination with known non-ligands as the negative samples of the training datasets. The collected post-2010 ligands and non-ligands were used as independent testing datasets for evaluating the performance of the developed VS models. These datasets are summarized in **Table** **1**.

The use of pre-2010 and post-2010 compounds as training and testing datasets was intended to mimic the case of VS models being developed in 2010 and subsequently tested a few years later against newly discovered compounds. In view that such training and testing datasets and their developed models may not be easily reproduced and comparatively evaluated, we designed alternative training and testing datasets by randomly separating all ligands and non-ligands of a receptor subtype into approximately 10 compound-sets, with 9 compound-sets as a training dataset and the remaining 1 as a testing dataset (these training and testing datasets contain similar number of compounds as the corresponding ones developed from pre-2010 and post-2010 compounds). There are 10 sets of training and testing datasets for each subtype with each of the 10 compound-sets used as a testing dataset once, all of which were tested in this work. These alternative datasets are summarized in **Table** **S1**.

Dopamine receptor subtype selective ligands have been discovered and evaluated based on the criterion that each ligand binds to a specific subtype with at least ∼10 fold higher binding affinity (Ki value) than that to another subtype [46]. Based on this criterion, we collected 97, 21, and 29 D1 selective ligands with >10 fold higher binding affinity over D2, D3 and D4 respectively, 43, 37 and 63 D2 selective ligands over D1, D3 and D4 respectively, 48, 99 and 85 D3 selective ligands over D1, D2 and D4 respectively, and 27, 408 and 207 D4 selective ligands over D1, D2 and D3 respectively (**Table** **2**). These subtype selective ligands were used as the positive samples to test subtype selectivity of our developed VS models.

**Table 2:** Datasets of our collected dopamine receptor D1, D2, D3 and D4 selective ligands against another subtype.

Dopamine receptor subtype	Selectivity against the second subtype	Number of subtype selective ligands against the second subtype	Range of binding affinity ratio	Mean of binding affinity ratio
D1	D2	97	10–4533	359
	D3	21	11–559	122
	D4	29	11–4600	770
D2	D1	43	10–3707	337
	D3	37	10–615	66
	D4	63	10–1851	113
D3	D1	48	17–38461	3863
	D2	99	10–6666	259
	D4	85	10–9111	950
D4	D1	27	13–4761	1315
	D2	408	10–10752	2962
	D3	207	10–51162	1175

The binding affinity ratio is the experimentally measured binding affinity to the second subtype divided by that to the first subtype: (Ki of the second subtype / Ki of the first subtype). This dataset was used as positive samples for testing subtype selectivity of our developed virtual screening models.

The binding subtypes of a number of multi-subtype dopamine ligands have been explicitly reported [16], [17]. These ligands and their binding subtypes were selected based on the criterion that they bind to each subtype with binding affinity Ki <1 μM. We collected 4 groups of dual-subtype ligands (147 D1–D2, 4 D1–D3, 8 D1–D4, and 100 D3-D4 ligands), 2 groups of triple-subtype ligands (39 D1–D2–D3 and 2 D1–D2–D3 ligands), and 1 group of quadruple-subtype ligands (60 D1–D2–D3–D4 ligands). Four of these groups with >10 ligands were selected as negative samples to test the ability of our developed VS models in predicting multi-subtype ligands (and thus the ability in separating subtype-selective and multi-subtype ligands) (**Table** **3**). There are three other groups with high numbers of multi-subtype ligands (569 D2–D3, 276 D2–D4 and 402 D2-D3-D4 ligands). Separation of these groups of multi-subtype ligands from the training datasets would significantly compromise the structural diversity of the training datasets. Therefore, these three groups were not removed from the training datasets. Inclusion of these groups in the training datasets does not enhance their subtype-selective signal. Instead they act as noise that tends to reduce the capability of the developed VS models in separating subtype-selective and multi-subtype ligands.

**Table 3:** Datasets of our collected dopamine receptor multi-subtype ligands.

Ligand Group	Binding Subtypes	Number of Ligands of Subtypes	Used as Testing Dataset
Dual Subtype Ligands	D1 and D2	147	Yes
	D1 and D3	4	No
	D1 and D4	8	No
	D3 and D4	100	Yes
Triple Subtype Ligands	D1, D2 and D3	39	Yes
	D1, D3 and D4	2	No
Quadruple Subtype Ligands	D1, D2, D3 and D4	60	Yes

Four groups of this dataset were used as negative samples for testing subtype selectivity of our developed multi-label machine learning models.

ERα and ERβ ligands were collected in the same manner as that of dopamine receptor ligands using keyword combinations of “estrogen”, “estrogen receptor”, “ER”, “ER alpha”, “ER beta”, “ligand”, “binding”, “binder”, “subtype selective”, and “selective ligand”. We collected 1146 ERα and 1234 ERβ ligands (with unknown status about their subtype selectivity or multi-subtype binding) and 761 and 786 ERα and ERβ non-ligands, which together with 64013 and 60603 putative ERα and ERβ non-ligands (generated by the same procedure as the putative dopamine receptor subtype non-ligands) were used for training 2BR-SVM VS models using the same procedure as that of the alternative dataset version of dopamine receptor subtype selective VS models. There are 10 sets of randomly assembled training and testing datasets for each estrogen receptor subtype with each of the 10 randomly generated compound-sets used as a testing dataset once, all of which were tested in this work. We also collected 40 and 55 ERα and ERβ selective ligands (with binding affinity ratios in the range of 10–2055 and 10–1143) and 63 ERα and ERβ multi-target ligands, which were used as independent testing datasets for testing the VS models. These datasets are summarized in **Table S2**.

### Molecular representations

The 2D structures of our collected compounds were drawn by using Chemdraw or from the ChEMBLdb [43] and Pubchem [23] databases. Each compound was represented by 98 molecular descriptors (**Table** **S3**) computed by using own developed MODEL program [47]. These 98 descriptors have been selected in our previous studies for developing VS models of a variety of target classes including GPCR ligands to screen large chemical libraries such as Pubchem compounds [18], [19], [48]. Although the structures of the binders of one target or subtype can be very different from those of another target or subtype, each binders set plus the representatives of the non-binders cover the same chemical space defined by the 13.56 million Pubchem compounds. Therefore, the same set of molecular descriptors was used in this work as well as our previous works [18], [48].

### Support vector machines

SVM is based on the structural risk minimization principle for minimizing both training and generalization error [49]. In linearly separable cases, SVM constructs a hyper-plane to separate active and inactive classes of compounds with a maximum margin. In nonlinearly separable cases, which frequently occur in classifying compounds of diverse structures [18], [19], [48], SVM maps the input vectors into a higher dimensional feature space by using the Radial Basis Function (RBF) kernel function. This kernel function has been extensively used and consistently shown better performance than other kernel functions [50], [51], [52]. In the high dimensional space, linear SVM can be applied for classifying the active and inactive compounds. For the parameters, a hard margin C=100000 was used and σ=0.4–0.6 were determined from 5 fold cross validation studies.

### Combinatorial SVM method

In combinatorial strategy, SVM models for each receptor subtype are separately constructed, which are subsequently used for parallel screening against each individual subtype to find compounds that only bind to one of the subtypes (putative subtype selective ligands) or simultaneously bind to multiple subtypes (putative subtype non-selective ligands) [18], [48].

### Two-step Binary relevance SVM method

Subtype selective ligands were selected by two steps. In the first step, a high performance SVM model was developed for each receptor subtype to select ligands of that subtype regardless of their selectivity towards other subtypes. The high performance in selecting ligands of a subtype was achieved by using comprehensive sets of known ligands and putative non-ligands of the corresponding receptor to train the respective SVM model [19]. In the second step, the Binary relevance (BR) method [30] was used for more refined selection of subtype selective ligands from the putative ligands selected in the first step. BR is a popular multiple binary classification method that transforms the original N-label dataset into N pairs of datasets with samples of each label as positive dataset and samples of the other N-1 labels as negative dataset [30].

### Multi-label K nearest neighbor method

ML-kNN implemented in the Mulan software package [30] was used in this work. ML-kNN [31] extends traditional kNN method to solve the multi-label problem. In the first step, ML-kNN classifies a compound ***x*** by linking it to the known ligand or non-ligand ***x*** *_i_* in the training dataset with closest Euclidean distance [53]. In the second step, statistical information, i.e. prior and posterior probabilities for the frequency of each label within the k nearest neighbors, is gained from the label sets of these neighboring ligands. In the third step, maximum a posteriori (MAP) principle is used to determine the label set for the unknown ligands. The default parameters in Mulan package were used in this work.

### The random k-labelsets decision tree method

RAkEL-DT implemented in the Mulan software package [30] was used in this work. The random k-labelsets (RAkEL) method [32] constructs an ensemble of label powerset (LP) classifiers. LP is a transformation method which considers each unique set of labels existed in multi-label training set as new single label. Since RAkEL is a transformation-based algorithm and it accepts a single-label classifier as a parameter, decision tree C4.5 algorithm was used for this purpose. C4.5 decision tree is a branch-test-based classifier [54]. A branch in a decision tree corresponds to a group of classes and a leaf represents a specific class. A decision node specifies a test to be conducted on a single attribute value, with one branch and its subsequent classes as possible outcomes of the test. C4.5 decision tree uses recursive partitioning to examine every attribute of the data and to subsequently rank them according to their ability to partition the remaining data, thereby constructing a decision tree. The default parameters in Mulan package were used in this work.

### Virtual screening model development, parameter determination and performance evaluation

All VS models for each dopamine receptor subtype were trained from the training datasets in **Table** **1**. The parameters were determined by 5-fold cross validation (CV) tests, and the performance of these VS models was evaluated by using the independent testing datasets in **Table** **1**. In each 5-fold CV test, the training dataset was divided into 5 groups of approximately equal number of positive samples and equal number of negative samples, with 4 groups used for training and 1 group used for testing the model. There are five such sets each with one unique group used as a testing set, from which five prediction models can be constructed. VS models were developed at different parameters. The parameters with the best overall 5-fold CV performance were selected for developing the final VS models.

The performance indicators can be derived from the numbers of true positives *TP* (true inhibitors), true negatives *TN* (true non-inhibitors), false positives *FP* (false inhibitors), and false negatives *FN* (false non-inhibitors). In 5-fold cross validation studies, the inhibitor and non-inhibitor prediction accuracies are given by sensitivity *SE*=*TP/(TP+FN*)*100 and specificity *SP*=TN/(TN+FP)*100 respectively. Prediction accuracies have also been frequently measured by overall prediction accuracy (*Q*) and Matthews correlation coefficient (*C*) [55].
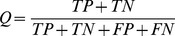
(1)

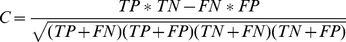
(2)


In the large database screening tests, the yield and false-hit rate are given by *TP/(TP+FN)* and *FP/(TP+FP*) respectively.

Determination of similarity level of a compound against dopamine receptor ligands in a dataset

The similarity level of a compound *i* with respect to the ligands of a dataset can be determined by using the Tanimoto coefficient *sim(i,j):* [56].
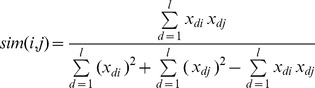
(3)


where *x_di_* represents a molecular fingerprint of compound *i* (there are 882 fingerprints calculated from the PaDEL-Descriptors program [57], *l* is the number of molecular fingerprints, *j* is the index of the ligand in the dataset most similar to compound *i*. The compound *i*. is assigned into one of the ten similarity levels based on its computed *sim(i,j)* values: 0.9–1, 0.8–0.9, 0.7–0.8, 0.6–0.7, 0.5–0.6, 0.4–0.5, 0.3–0.4, 0.2–0.3, 0.1–0.2, and 0–0.1. Compounds are typically considered to be highly similar if *sim(i,j)* is no less than 0.8 or 0.9 [58], [59].

### Determination of dopamine receptor subtype selective features by feature selection method

Molecular features important for dopamine receptor subtype selective ligands were probed by using a feature selection method, recursive feature elimination (RFE) method, extensively used in selecting molecular features of compounds of specific pharmacodynamic and pharmacokinetic properties 60]. In this approach, the level of contribution of individual molecular descriptor to SVM classification of ligands of a subtype against ligands of other subtypes was ranked and the top-ranked ones were selected based on the evaluation of the variation of the SVM objective function *J* caused by the removal of an individual descriptor 61]. The variation *DJ*(*i*) due to the removal of a descriptor *i* is computed by 
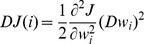
 with the weight variation determined by *Dwi = wi*. In this work, Gaussian kernels were used for developing SVM models. In this case, 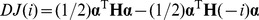, where *H* is the matrix with elements *y_ i_ y_ j_* exp(-||*x_i_* - *x_j_*||^ 2^/(2*σ* ^2^)), *H*(-*i*) is the matrix computed by the same method as matrix *H* but with its i-th component removed, *y_ i_* is the vector composed of molecular descriptors, *1* is an *m* dimensional identity vector (*m* is the number of compounds in a training dataset), and the component of vector α is kept in the range of 0 ≤ α_ k_ ≤ C.

The computational procedure for selecting subtype selective features is as follows: For a specific subtype, the corresponding SVM model developed in the second step of the 2SBR-SVM method is processed by iteratively evaluating and eliminating molecular descriptors at different parameter σ values based on 5-fold cross-validation. In the first step, for a fixed σ, the SVM is trained by using the complete set of descriptors (feature set). The second step is to compute the ranking criterion score *DJ*(*i*) for every existing descriptor. All the computed *DJ*(*i*) is then ranked in descending order. The third step is to remove the m descriptors with smallest criterion scores (m=4 in this work). In the fourth step, the SVM is retrained by using the remaining molecular descriptors and a new prediction accuracy of 5-fold cross-validation is computed. The second to fourth steps are repeated for multiple-iterations until all descriptors are removed. For another fixed σ, the first to fourth steps are repeated.

## Results and Discussion

### 5-fold cross-validation tests

The results of 5-fold CV tests of the SVM models of D1, D2, D3 and D4 ligands are shown in **Table S4**. Overall, the sensitivity, specificity, overall accuracy and the Matthews correlation coefficients of the best performing SVM models are in the range of 87.8%–95.3%, 99.6%–99.9%, 99.3%–99.8%, and 0.74–0.90 respectively. These results are comparable to those of our earlier studies [48], indicating that the SVM models for dopamine receptor subtypes have similar prediction capability as those for other target classes. The VS models with the best 5–fold CV performance were further tested on independent sets of dopamine receptor ligands and non-ligands published since 2010 and not in the training datasets, which are also shown in **Table S4**. The sensitivity, specificity and overall accuracy are in the range of 71.2%–89.7%, 61.5%–76.0% and 71.4%–82.7% respectively. The sensitivity is substantially smaller than that of 5–fold CV tests. This is because many of the post–2010 ligands in the independent datasets are structurally different from those of the pre-2010 ligands in the training datasets. As shown in **Table** **1**, 16.3%–34.5% of the post–2010 ligands are outside the chemical families of pre-2010 ligands in the training datasets. The specificity is also significantly smaller than that of the 5-fold CV tests. This is partly because many non-ligands have weak (Ki 10–50 μM) binding activity and may thus be difficult to be separated from the ligands.

The VS performance of the SVM VS models developed by the 10 sets of alternative training and testing datasets is provided in **Table** **S1**. The sensitivity, specificity, overall accuracy and the Matthews correlation coefficients of these SVM models in classifying dopamine receptor subtype ligands and non-ligands are in the range of 79.1%–94.8%, 99.6%–99.9%, 99.3%–99.9%, and 0.73–0.90 respectively, which are very similar to those of the SVM models developed by pre–2010 and tested by post-2010 compounds. A further analysis of structures of the randomly assembled datasets and those of the chronologically assembled datasets showed that most of the active and inactive scaffolds are mutually represented on both sides because of the significant structural diversity in these datasets. Therefore, the VS performance of SVM models developed by chronologically assembled datasets can be compared with those models developed by using datasets assembled by conventional approach.

### Applicability domains of the developed SVM VS models

Our SVM VS models for each dopamine receptor subtype were developed by using known ligands and non-ligands of the subtype, and the putative non-ligands composed of representative compounds of all of the compound families in the Pubchem chemical space that contain no known ligand of the subtype. Theoretically, these VS models are expected to be applicable in the chemical space defined by the known ligands, known non-ligands, and the 13.56 M Pubchem compounds. If this is true, in addition to good predictive performance on the known ligands, these VS models are expected to consistently identify very small percentages of Pubchem compounds as subtype selective ligands regardless of their similarity levels to the known ligands. Alternatively, if the applicability domain of these models covers limited chemical space around known ligands, then the number of identified Pubchem compounds may increase substantially beyond the applicability domain (i.e. at lower similarity levels). To determine the applicability domain of each SVM VS model, we divided 13.56 M PubChem compounds into groups of 10 similarity levels with respect the known ligands of each receptor subtype (defined in the methods section), and then monitored if the number of the SVM identified PubChem compounds significantly increases at higher similarity levels. As shown in **Table** **S5**, the percentages of identified Pubchem compounds for all four receptor subtypes (0.0489%–0.0521% for D1, 0.131%–0.135% for D2, 0.143%–0.147% for D3, and 0.157%–0.160% for D4 respectively) are consistently small and show little variations at different similarity levels. This suggests that the applicability domains of our SVM VS models likely cover the chemical space defined by the known ligands, known non-ligands and the PubChem compounds.

### Prediction performance on dopamine receptor subtype selective and multi-subtype ligands

The performance of our new method 2SBR-SVM and that of the three previously used methods Combi-SVM, ML-kNN and RAkEL-DT in predicting dopamine subtype selective ligands was determined as follows: For each set of dopamine receptor subtype selective ligands against another subtype, the developed VS model of the subtype and that of the second subtype were both used to screen these ligands. The percentage of these ligands selected by the first model but not by the second model was used to measure the performance of the VS models in selecting subtype selective ligands. The relevant results are shown in **Table** **4**.

**Table 4:** The performance of our new method 2SBR-SVM and that of previously used methods Combi-SVM, ML-kNN and RAkEL-DT in predicting dopamine receptor subtype selective ligands.

			Percent of subtype selective ligands predicted as subtype selective with respect to the second subtype			
Dopamine receptor subtype	Selectivity against the second subtype	Number of subtype selective ligands	Combi-SVM	ML-kNN	RAkEL-DT	2SBR-SVM
D1	D2	97	13.40%	30.93%	75.26%	86.60%
	D3	21	23.81%	23.81%	47.62%	66.67%
	D4	29	17.24%	58.62%	44.83%	65.52%
	**average**		18.15%	37.79%	55.90%	72.93%
D2	D1	43	55.81%	62.79%	69.77%	93.02%
	D3	37	16.22%	21.62%	62.16%	81.08%
	D4	63	14.29%	39.68%	30.16%	82.54%
	**average**		28.77%	41.36%	54.03%	85.55%
D3	D1	48	72.92%	87.50%	85.42%	56.25%
	D2	99	22.22%	26.26%	50.51%	51.52%
	D4	85	17.65%	31.76%	22.35%	50.59%
	**average**		37.60%	48.51%	52.76%	52.79%
D4	D1	27	74.07%	70.37%	85.19%	82.50%
	D2	408	33.33%	28.43%	57.60%	88.00%
	D3	209	26.79%	24.40%	45.46%	83.73%
	**average**		44.73%	41.07%	62.75%	84.74%

As shown in **Table 4**, the three previously used methods showed mostly moderate and in minority cases good performance in predicting dopamine receptor subtype selective ligands. Specifically, 13.4%–23.8%, 14.3%–55.8%, 17.7%–77.9% and 26.8%–74.1% of the D1, D2, D3 and D4 selective ligands were correctly predicted by Combi-SVM as subtype selective ones. ML-kNN showed better performance, correctly predicting 23.8%–58.6%, 21.6%–62.8%, 26.3%–87.5% and 24.4%–70.4% of the D1, D2, D3 and D4 selective ligands as subtype selective ones. The RAkEL–DT method achieved the best performance among the three methods, correctly predicting 44.8%–75.3%, 30.2%–69.8%, 22.4%–85.4% and 45.5%–85.2% of the D1, D2, D3 and D4 selective ligands as subtype selective ones. On the other hand, our new method 2BR–SVM produced significantly improved performance, correctly predicting 66.5%–86.6%, 81.1%–93.0%, 50.6%–56.3% and 82.5%–88.0% of the D1, D2, D3 and D4 selective ligands as subtype selective ones. This suggests that our two–step strategy with one step focusing on subtype binding and another on selectivity works more effectively than the three previously used methods in predicting dopamine receptor subtype selective ligands.

The improved subtype selective performance of the 2BR-SVM method arises from its more rigorous evaluation of minor structural and physicochemical differences of subtype selective ligands. Comparative structural analysis has shown that some D2 selective and D3 selective ligands are highly similar in structure and interact with their respective subtypes in a very similar binding mode with some functional group adopting different orientation at sites of non-conserved residues [46]. Such minor differences may not be adequately distinguished by conventional VS models developed by training datasets with inadequate representation of ligands of other subtypes, but may be distinguished by 2BR-SVM method with additional models developed by training datasets with sufficient representation of other subtypes.

The performance in predicting dopamine subtype selective ligands is measured not only by the capability in selecting subtype selective ligands, but also on the ability in differentiating them from multi-subtype ligands. Good prediction on subtype selective ligands needs to be complemented by equally good performance in predicting multi-subtype ligands as subtype non-selective ones. This performance was determined as follows: For each set of multi-subtype ligands (e.g. triple-subtype D1, D2 and D3 ligands), the VS models of all of the corresponding subtypes (e.g. D1, D2 and D3) were used to screen the multi-subtype ligands in the set. The percentage of these ligands selected by the model of more than one subtype was used to measure the performance of the VS models in predicting multi-subtype ligands as subtype non-selective ligands. The results are shown in **Table 5**.

**Table 5:** The performance of our new method 2SBR-SVM and that of previously used methods Combi-SVM, ML-kNN and RAkEL-DT in predicting dopamine receptor multi-subtype ligands as non-selective ligands.

			Percent of multi-subtype ligands predicted as non-selective ligands			
Ligand Group	Binding subtypes	Number of Multi- Subtype Ligands	Combi-SVM	ML-kNN	RAkEL-DT	2SBR-SVM
Dual Subtype Ligands	D1 and D2	147	68.02%	31.97%	35.37%	76.19%
	D3 and D4	100	83.0%	37.0%	39.0%	81.0%
Triple Subtype Ligands	D1, D2 and D3	39	76.92%	28.2%	33.33%	71.79%
Quadruple Subtype Ligands	D1, D2, D3 and D4	60	75.42%	36.67%	38.75%	71.67%

Of the three previously used methods, Combi-SVM showed the best performance in predicting dopamine receptor multi-subtype ligands as subtype non-selective ones, correctly predicting 68.0%, 83.0%, 76.9% and 75.4% of the D1-D2, D3-D4, D1-D2-D3 and D1-D2-D3-D4 multi-subtype ligands as subtype non-selective ones. On the other hand, only 32.0%, 37.0%, 28.2% and 36.7% of the D1-D2, D3-D4, D1-D2-D3 and D1-D2-D3-D4 multi-subtype ligands were predicted by ML-kNN as subtype non-selective ones, and only 35.4%, 39.0%, 33.3% and 38.8% of the D1-D2, D3-D4, D1-D2-D3 and D1-D2-D3-D4 multi-subtype ligands were predicted by RAkEL-DT as subtype non-selective ones. Hence, the better performance of ML-kNN and RAkEL-DT over Combi-SVM in predicting subtype selective ligands is off-set by the poorer performance in predicting multi-subtype ligands as subtype non-selective. Taken these two indicators together, Combi-SVM appears to show better overall performance in predicting subtype selective and subtype non-selective ligands than the ML-kNN and RakEL-DT methods.

The performance of our new method 2SBR-SVM in predicting dopamine receptor subtype non-selective ligands is similar to that of Combi-SVM, correctly predicting 76.2%, 81.0%, 71.8% and 71.7% of the D1-D2, D3-D4, D1-D2-D3 and D1-D2-D3-D4 multi-subtype ligands as subtype non-selective ones. Thus, our new method maintains the same performance level as that of the best performing method of the previously used methods in predicting dopamine receptor subtype non-selective ligands. The lack of improvement by our new method in predicting dopamine receptor subtype non-selective ligands may be partly due to the quality of training datasets. It is noted that three groups of multi-subtype ligands were included as positive samples in the training datasets, which likely affect the ability of the SVM models in predicting multi-subtype ligands as subtype non-selective ones.

### Virtual screening performance in searching large chemical libraries

The virtual screening performance of our new method 2SBR-SVM and our previously developed method Combi-SVM was evaluated by using them to screen 13.56 M Pubchem compounds, 168,016 MDDR compounds and 657,736 ChEMBLdb compounds to determine the numbers of Pubchem, MDDR, and ChEMBLdb compounds predicted as D1, D2, D3 and D4 selective ligands, which are shown in **Table** **6**. For comparison, **Table** **6** also includes the results of SVM (single label) in identifying Pubchem compounds as putative D1, D2, D3 and D4 ligands regardless of their possible binding with another subtype. In screening Pubchem compounds, the number of D1, D2, D3 and D4 selective virtual hits identified by 2SBR-SVM and the corresponding virtual hit rate is 650 and 0.0048%, 1132 and 0.0083%, 1498 and 0.011%, and 1961 and 0.015% respectively, which is significantly smaller than those identified by Combi-SVM. The number of D1, D2, D3 and D4 selective virtual hits identified by Combi-SVM and the corresponding virtual hit rate is 4948 and 0.037%, 10080 and 0.074%, 6055 and 0.045%, and 9180 and 0.068% respectively. The number of virtual hits identified by Combi-SVM is nonetheless substantially smaller than that of single label SVM. The number of D1, D2, D3 and D4 selective virtual hits identified by single label SVM and the corresponding virtual hit rate is 6798 and 0.05%, 17786 and 0.13%, 19813 and 0.15%, and 21444 and 0.16% respectively. Some of the identified virtual hits are possible subtype selective ligands. Therefore the true false hit rates of the tested VS models are likely smaller than the computed virtual hit rates. The false hit rates of 2SBR-SVM in screening 13.56 million Pubchem compounds can then be estimated as ≤0.0048%, ≤0.0083%, ≤0.011% and ≤0.015% for D1, D2, D3 and D4 selective ligands respectively. Therefore, 2SBR-SVM produced very low false hit rates in screening large chemical libraries as well as good performance in selecting subtype selective ligands.

**Table 6:** Virtual screening performance of our new method 2SBR-SVM and that of our previously used method Combi-SVM in scanning 168,016 MDDR compounds and 657,736 ChEMBLdb compounds, and 13.56 million Pubchem compounds.

Dopamine receptor subtype	Method	Number and Percent of the 13.56M PubChem Compounds Identified as subtype selective ligands	Number and Percent of the 168,016 MDDR Compounds Identified as subtype selective ligands	Number and Percent of the 657,736 ChemBLdb Compounds Identified as subtype selective ligands
D1	SVM (Single Label)	6798(0.0501%)	463(0.28%)	1034(0.16%)
	Combi-SVM	4948(0.0365%)	383(0.23%)	755(0.11%)
	2SBR-SVM	650(0.0048%)	140(0.08%)	355(0.05%)
D2	SVM (Single Label)	17786(0.1312%)	1105(0.66%)	3208(0.49%)
	Combi-SVM	10080(0.0743%)	712(0.42%)	2023(0.31%)
	2SBR-SVM	1132(0.0083%)	108(0.06%)	686(0.10%)
D3	SVM (Single Label)	19813(0.1461%)	1149(0.68%)	3057(0.46%)
	Combi-SVM	6055(0.0447%)	679(0.40%)	1894(0.29%)
	2SBR-SVM	1498(0.0110%)	156(0.09%)	687(0.10%)
D4	SVM (Single Label)	21444(0.1581%)	1160(0.69%)	3489(0.53%)
	Combi-SVM	9186(0.0677%)	790(0.47%)	2579(0.39%)
	2SBR-SVM	1961(0.0145%)	134(0.08%)	907(0.14%)

For comparison, the results of single label SVM, which identify putative subtype binding ligands regardless of their possible binding to another subtype, are also included.

As shown in **Table 6**, in screening MDDR and ChEMBLdb compounds, 2SBR-SVM as well as Combi-SVM and single label SVM produced reasonably low virtual hit rates that are in the range of 0.06%–0.09% and 0.05%–0.14% respectively, which are 10 fold higher than those in screening Pubchem compounds. MDDR and ChEMBLdb compounds as a collection of bioactive agents tend to be structurally closer to the dopamine receptor ligands than many Pubchem compounds that consist of high percentage of inactive compounds. Therefore, it tends to be more difficult for 2SBR-SVM to distinguish dopamine receptor ligands from some of the non-ligands in MDDR and ChEMBLdb databases, leading to higher virtual-hit rates. The virtual hit rates of 2SBR-SVM in screening MDDR and ChEMBLdb compounds are substantially (2–10 fold) smaller than those of Combi-SVM and single label SVM, which suggests that 2SBR-SVM is capable of achieving lower false-hit rate in screening bioactive compounds than more conventional SVM methods.

Although it is unclear how many true D1, D2, D3 and D4 selective ligands are contained in Pubchem database. Some crude estimates can be made. As shown in **Table** **1** and **Table** **2**, the number of known ligands of a dopamine receptor subtype is in the range of 550–2337, and the number of known dopamine receptor subtype selective ligands is in the range of 21–408. The known subtype selective ligands are approximately 10 fold less in numbers than the known ligands of a subtype. While the numbers of the published D1, D2, D3, and D4 ligands continuously increase through the years (**Figure S1, S2, S3 and S4**), there are signs of significant reduction of the growth rates at the level of 2000–3000 ligands. These trends tend to project the existence of no more than several thousand undiscovered ligands for each dopamine receptor subtype in the chemical space defined by the Pubchem, MDDR and ChEMBLdb compounds. Hence, the number of subtype selective virtual hits identified by 2SRB-SVM is closer to the estimated upper limit of undiscovered dopamine receptor subtype ligands than those of Combi-SVM and single label SVM.

### Dopamine receptor subtype selective features

The molecular descriptors important for distinguishing the ligands of every dopamine receptor subtype and the ligands of other subtypes were determined by using the feature selection method [60] outlined in the method section, which are provided in **Table** **7**. The top-ranked D1 selective descriptors are number of O atoms, sum of Estate of atom type dssC, ssO and ssNH, graph-theoretical shape coefficient, and sum of H Estate of atom type HsNH2. These descriptors are consistent with the D1 selective features derived from a pharmacophoric model that includes positive nitrogens (linked to ssNH, HsNH2), hydrogen bond acceptor (linked to O, ssO) and donor (linked to ssNH, HsNH2) [14]. The top-ranked D2 selective descriptors are number of H-bond acceptor, sum of H Estate of atom types HaaNH and HCsats, and sum of Estate of atom type dssC, aasC and aaNH. These are consistent with a CoMSIA based analysis that suggests that D2 selectivity is determined by hydrogen bond acceptor (linked to H-bond acceptor) and donor (linked to HaaNH), hydrophobic (linked to HCsats, dssC, aasC), and electrostatic (linked to HaaNH, aaNH) factors [10]. These are also consistent with the conclusion from a pharmacophoric model that two hydrogen acceptors or one hydrogen acceptor plus one donor are critically important for D2 selectivity of some ligands [14].

**Table 7:** Top-ranked molecular descriptors for distinguishing dopamine receptor subtype D1, D2, D3 or D4 selective ligands selected by RFE feature selection method.

Dopamine receptor subtype	Top-ranked molecular descriptors for distinguishing subtype selective ligands and ligands of other subtypes
D1	Number of O atoms, Sum of Estate of atom type dssC, Sum of Estate of atom type ssO, Sum of Estate of atom type ssNH, Graph-theoretical shape coefficient, Sum of H Estate of atom type HsNH2
D2	Number of H-bond acceptor, Sum of H Estate of atom type HaaNH, Sum of H Estate of atom type HCsats, Sum of Estate of atom type dssC, Sum of Estate of atom type aasC, Sum of Estate of atom type aaNH
D3	Sum of Estate of atom type dsCH, Sum of H Estate of atom type HsOH, Sum of H Estate of atom type HCsats, Sum of Estate of atom type aaaC, Sum of Estate of atom type sOH, Number of H-bond donnor
D4	Molecular path count of length 2, Sum of Estate of atom type ssCH2, 3th order Kier shape index, Topological radius, Sum of Estate of atom type aasC, Kier Molecular Flexibility Index

The top-ranked D3 selective descriptors are sum of Estate of atom type dsCH, aaaC and sOH, sum of H Estate of atom type HsOH and HCsats, and number of H-bond donor. These are consistent with the conclusions from several CoMSIA models that correlate D3 selectivity with specific hydrogen bond donor (linked to H-bond donor, sOH, HsOH), hydrophobic (linked to dsCH, aaaC), and electrostatic (linked to sOH, HsOH) factors [10], [15]. Moreover, a study of a D3 selective ligand further shows that hydrogen bonding from a hydroxyl group is important for conferring D3 selectivity [10]. The top-ranked D4 selective descriptors are molecular path count of length 2, sum of Estate of atom type ssCH2 and aasC, 3th order Kier shape index, topological radius, and Kier molecular flexibility index. These are consistent with a report that D4 selectivity is strongly influenced by the geometry and orientation of specific chemical groups (linked to molecular path count of length 2, 3th order Kier shape index, topological radius, and Kier molecular flexibility index) [9]. The consistency of our selected molecular descriptors and the literature-reported features for D1, D2, D3, and D4 selectivity suggests that the subtype selective molecular descriptors selected by our feature selection method may be potentially useful for facilitating the design or search of dopamine subtype selective ligands.

### Virtual screening performance of the two-step binary relevance SVM method in searching estrogen receptor subtype selective ligands

The VS performance of the SVM models for each ER subtype developed by the 10 sets of randomly assembled training and testing datasets is provided in **Table** **S2**. The sensitivity, specificity, overall accuracy and the Matthews correlation coefficients of these SVM models in classifying ER subtype ligands and non-ligands are in the range of 92.9%–97.6%, 99.7%–99.9%, 99.7%–99.9%, and 0.84–0.92 respectively, which are very similar to those of the dopamine receptor subtype. Moreover, as shown in **Table S6 and** **S7**, the performance of 2SBR-SVM in identifying ERα selective ligands (85.0%), ERβ selective ligands (80.0%), ERα and ERβ multi-subtype ligands (69.8%), and in screening Pubchem, MDDR and ChEMBLdb compounds (virtual hit rates 0.0094%–0.0104%, 0.056%–0.064%, and 0.033%–0.034%) is at very similar levels as those of the dopamine receptor subtype. Therefore, our 2BR-SVM method is likely applicable to different receptor-ligand systems.

### Conclusion

Virtual screening methods have been increasingly explored for facilitating the discovery of target selective drugs for enhanced therapeutics and reduced side effects. Our study further suggested that the two-step target binding and selectivity support vector machines virtual screening tools developed from protein subtype ligands with unspecified subtype selectivity are capable of identifying protein subtype selective ligands at good yields, subtype selectivity and low false-hit rates in screening large chemical libraries. Our method may be combined with other virtual screening methods [62], [63], [64], [65], [66], [67], [68] to facilitate more effective and efficient search of novel subtype selective drug leads from larger chemical libraries. The capability of virtual screening tools can be further enhanced by the incorporation of the knowledge of existing and newly discovered subtype selective [2], [4] and multi-subtype [16], [17] ligands, and by the further improvement of virtual screening algorithms and parameters [19], [69], [70], [71], [72], [73], [74].

## Supporting Information

Figure S1 **Number of published D1 receptor ligands from 1980 to present.**
(DOC)

Figure S2 **Number of published D2 receptor ligands from 1987 to present.**
(DOC)

Figure S3 **Number of published D3 receptor ligands from 1980 to present.**
(DOC)

Figure S4 **Number of published D4 receptor ligands from 1980 to present**.
(DOC)

Table S1 **Statistics of alternative training and testing datasets for D1, D2, D3 and D4 subtypes, and the performance of SVM models developed and tested by these datasets in predicting D1, D2, D3 and D4 ligands. SE, SP, Q and C are sensitivity, specificity, overall accuracy and Matthews correlation coefficient respectively.**
(DOC)

Table S2 **Statistics of the randomly assembled training and testing datasets for ERα and ERβ, and the performance of SVM models developed and tested by these datasets in predicting ERα and ERβ ligands. SE, SP, Q and C are sensitivity, specificity, overall accuracy and Matthews correlation coefficient respectively.**
(DOC)

Table S3 **List of 98 molecular descriptors computed by using our own developed MODEL program.**
(DOC)

Table S4 **Results of 5-fold cross validation (CV) tests of SVM models in predicting D1, D2, D3 and D4 ligands.** SE, SP, Q and C are sensitivity, specificity, overall accuracy and Matthews correlation coefficient respectively.
(DOC)

Table S5 **Numbers of Pubchem compounds at different similarity levels with respect to known ligands of each dopamine receptor subtype, and percent of these compounds identified by SVM VS model as subtype selective ligands.**
(DOC)

Table S6 The performance of our new method 2SBR-SVM and that of previously used methods Combi-SVM, ML-kNN and RAkEL-DT in predicting estrogen receptor subtype selective and multi-subtype ligands.
(DOC)

Table S7 **Virtual screening performance of our new method 2SBR-SVM and that of our previously used method Combi-SVM in scanning 13.56 million Pubchem compounds, 168,016 MDDR compounds and 657,736 ChEMBLdb compounds.** For comparison, the results of single label SVM, which identify putative subtype binding ligands regardless of their possible binding to another subtypes, are also included.
(DOC)
