# Supplementary figures and images for: A Two-Step Target Binding and Selectivity Support Vector Machines Approach for Virtual Screening of Dopamine Receptor Subtype-Selective Ligands
Source: PLoS One. 2012 Jun 15;7(6):e39076. doi: 10.1371/journal.pone.0039076 (PMC3376116; doi:10.1371/journal.pone.0039076)

**Supplementary Figure S1** Number of published D1 receptor ligands from 1980 to present.


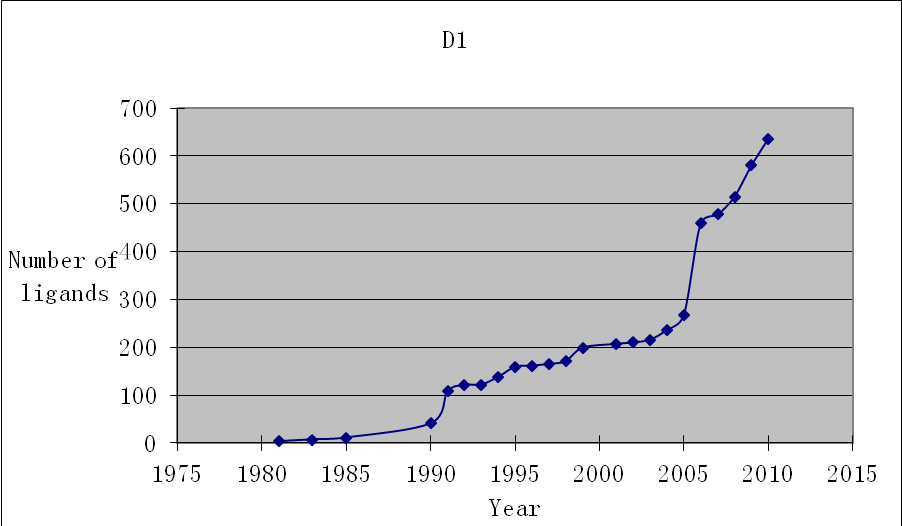

Supplement: Figure S1 — Number of published D1 receptor ligands from 1980 to present. (DOC) [file pone.0039076.s001.doc]

**Supplementary Figure S2** Number of published D2 receptor ligands from 1987 to present.


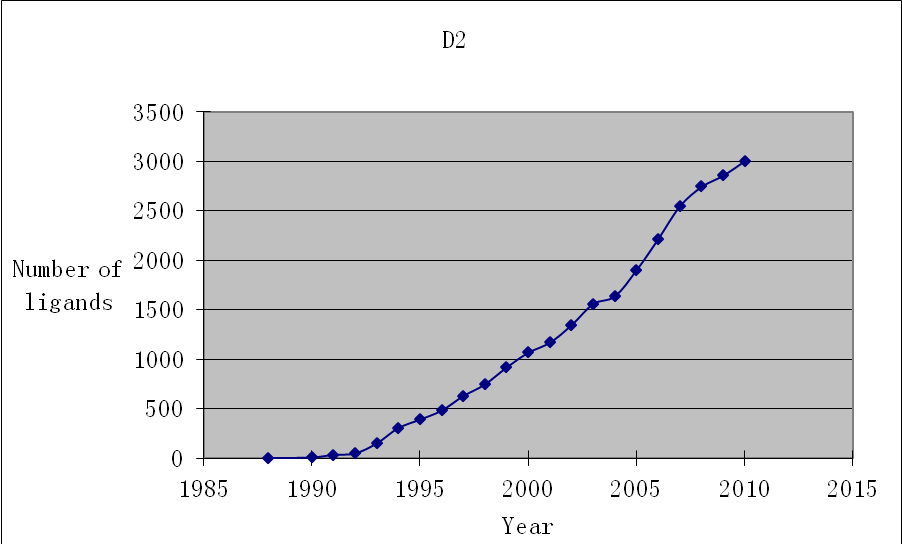

Supplement: Figure S2 — Number of published D2 receptor ligands from 1987 to present. (DOC) [file pone.0039076.s002.doc]

**Supplementary Figure S3** Number of published D3 receptor ligands from 1980 to present.


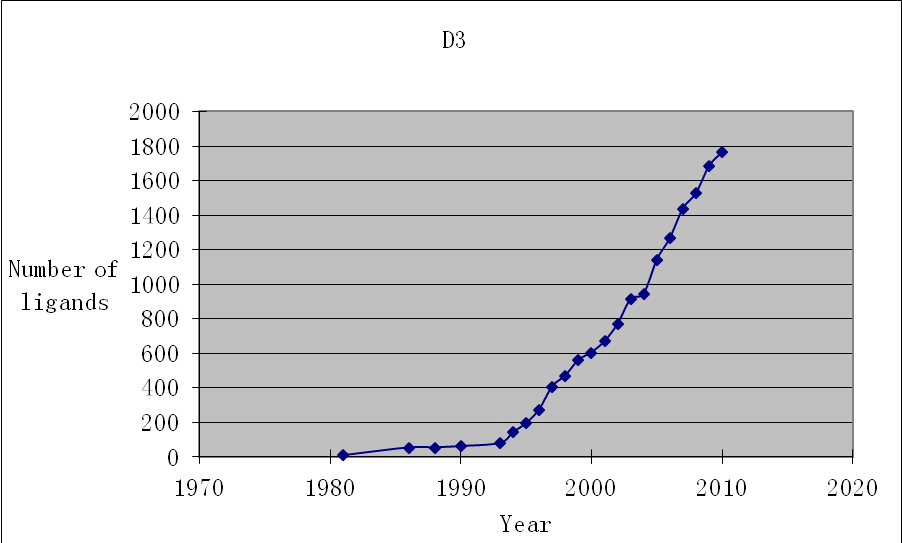

Supplement: Figure S3 — Number of published D3 receptor ligands from 1980 to present. (DOC) [file pone.0039076.s003.doc]

**Supplementary Figure S4** Number of published D4 receptor ligands from 1980 to present.


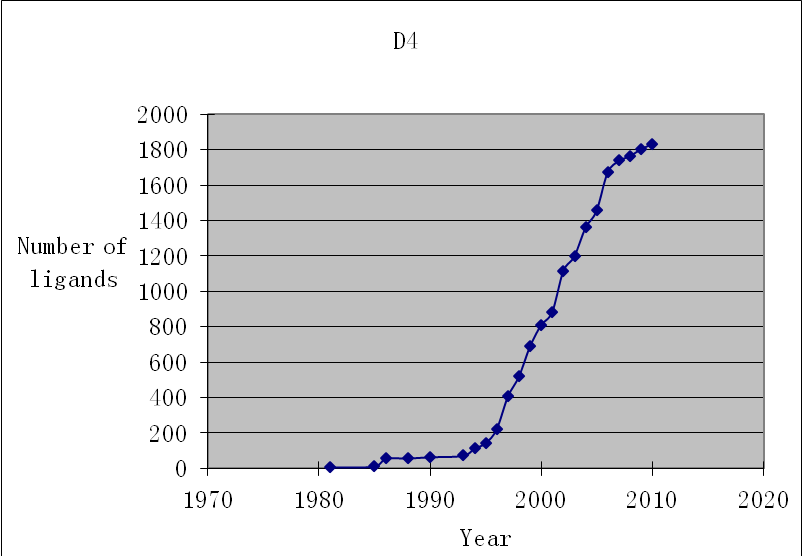

Supplement: Figure S4 — Number of published D4 receptor ligands from 1980 to present. (DOC) [file pone.0039076.s004.doc]
