# Supplementary material for: A Two-Step Target Binding and Selectivity Support Vector Machines Approach for Virtual Screening of Dopamine Receptor Subtype-Selective Ligands
Source: PLoS One. 2012 Jun 15;7(6):e39076. doi: 10.1371/journal.pone.0039076 (PMC3376116; doi:10.1371/journal.pone.0039076)
Supplement: Table S1 — Statistics of alternative training and testing datasets for D1, D2, D3 and D4 subtypes, and the performance of SVM models developed and tested by these datasets in predicting D1, D2, D3 and D4 ligands. SE, SP, Q and C are sensitivity, specificity, overall accuracy and Matthews correlation coefficient respectively. (DOC) [file pone.0039076.s005.doc]

**Supplementary Table S1** Statistics of alternative training and testing datasets for D1, D2, D3 and D4 subtypes, and the performance of SVM models developed and tested by these datasets in predicting D1, D2, D3 and D4 ligands. SE, SP, Q and C are sensitivity, specificity, overall accuracy and Matthews correlation coefficient respectively.

| **Dopamine Receptor Subtype** | **Alternative dataset** | **Number of ligands/non-ligands in alternative training and testing dataset** | **VS performance on testing dataset** | | | |
| --- | --- | --- | --- | --- | --- | --- |
| D1 |  |  | **SE** | **SP** | **Q** | **C** |
|  | 1 | 441/58914 and 50/6546 | 92.00% | 99.89% | 99.83% | 0.79 |
|  | 2 | 443/58914 and 48/6546 | 81.25% | 99.93% | 99.80% | 0.73 |
|  | 3 | 443/58914 and 48/6546 | 91.66% | 99.93% | 99.87% | 0.84 |
|  | 4 | 443/58914 and 48/6546 | 79.10% | 99.95% | 99.80% | 0.73 |
|  | 5 | 443/58914 and 48/6546 | 89.58% | 99.89% | 99.81% | 0.77 |
|  | 6 | 442/58914 and 49/6546 | 91.84% | 99.98% | 99.92% | 0.9 |
|  | 7 | 442/58914 and 49/6546 | 93.88% | 99.90% | 99.86% | 0.83 |
|  | 8 | 442/58914 and 49/6546 | 91.84% | 99.98% | 99.92% | 0.9 |
|  | 9 | 442/58914 and 49/6546 | 89.80% | 99.98% | 99.91% | 0.88 |
|  | 10 | 442/58914 and 49/6546 | 93.88% | 99.92% | 99.88% | 0.84 |
|  | **AVE** |  | 89.48% | 99.94% | 99.86% | 0.82 |
|  | **S.D** |  | 0.05127 | 0.00036 | 0.00048 | 0.06402 |
|  | **S.E.M** |  | 0.01621 | 0.00011 | 0.00015 | 0.02025 |
| D2 | **Alternative dataset** | **Number of ligands/non-ligands in alternative training and testing dataset** | **SE** | **SP** | **Q** | **C** |
|  | 1 | 2178/58914 and 242/6546 | 88.84% | 99.71% | 99.32% | 0.81 |
|  | 2 | 2178/58914 and 242/6546 | 94.24% | 99.79% | 99.59% | 0.88 |
|  | 3 | 2178/58914 and 242/6546 | 93.00% | 99.74% | 99.50% | 0.86 |
|  | 4 | 2178/58914 and 242/6546 | 93.42% | 99.77% | 99.54% | 0.87 |
|  | 5 | 2178/58914 and 242/6546 | 91.82% | 99.58% | 99.33% | 0.8 |
|  | 6 | 2178/58914 and 242/6546 | 90.91% | 99.74% | 99.42% | 0.84 |
|  | 7 | 2178/58914 and 242/6546 | 94.21% | 99.71% | 99.51% | 0.86 |
|  | 8 | 2178/58914 and 242/6546 | 89.67% | 99.71% | 99.35% | 0.82 |
|  | 9 | 2178/58914 and 242/6546 | 89.67% | 99.68% | 99.32% | 0.81 |
|  | 10 | 2178/58914 and 242/6546 | 92.56% | 99.74% | 99.48% | 0.86 |
|  | **AVE** |  | 91.83% | 99.72% | 99.44% | 84.10% |
|  | **S.D** |  | 0.01973 | 0.00058 | 0.00101 | 0.02885 |
|  | **S.E.M** |  | 0.00624 | 0.00018 | 0.00032 | 0.00912 |
| D3 | **Alternative dataset** | **Number of ligands/non-ligands in alternative training and testing dataset** | **SE** | **SP** | **Q** | **C** |
|  | 1 | 1215/57564 and 135/6356 | 93.33% | 99.78% | 99.64% | 0.83 |
|  | 2 | 1215/57564 and 135/6356 | 92.59% | 99.79% | 99.65% | 0.83 |
|  | 3 | 1215/57564 and 135/6356 | 91.85% | 99.79% | 99.63% | 0.83 |
|  | 4 | 1215/57564 and 135/6356 | 91.11% | 99.80% | 99.63% | 0.82 |
|  | 5 | 1215/57564 and 135/6356 | 91.11% | 99.85% | 99.68% | 0.84 |
|  | 6 | 1215/57564 and 135/6356 | 93.33% | 99.85% | 99.72% | 0.87 |
|  | 7 | 1215/57564 and 135/6356 | 92.59% | 99.83% | 99.68% | 0.85 |
|  | 8 | 1215/57564 and 135/6356 | 90.37% | 99.80% | 99.60% | 0.81 |
|  | 9 | 1215/57564 and 135/6536 | 94.81% | 99.86% | 99.75% | 0.88 |
|  | 10 | 1215/57564 and 135/6536 | 94.81% | 99.88% | 99.78% | 0.89 |
|  | **AVE** |  | 92.59% | 99.82% | 99.68% | 84.50% |
|  | **S.D** |  | 0.01521 | 0.00035 | 0.00058 | 0.02677 |
|  | **S.E.M** |  | 0.00481 | 0.00011 | 0.00018 | 0.00847 |
| D4 | **Alternative dataset** | **Number of ligands/non-ligands in alternative training and testing dataset** | **SE** | **SP** | **Q** | **C** |
|  | 1 | 1332/57920 and 148/6380 | 93.91% | 99.62% | 99.49% | 0.80 |
|  | 2 | 1332/57920 and 148/6380 | 93.24% | 99.69% | 99.54% | 0.81 |
|  | 3 | 1332/57920 and 148/6380 | 91.89% | 99.78% | 99.60% | 0.83 |
|  | 4 | 1332/57920 and 148/6480 | 91.89% | 99.74% | 99.57% | 0.82 |
|  | 5 | 1332/57920 and 148/6480 | 91.89% | 99.80% | 99.63% | 0.84 |
|  | 6 | 1332/57920 and 148/6480 | 92.57% | 99.89% | 99.72% | 0.87 |
|  | 7 | 1332/57920 and 148/6480 | 91.21% | 99.91% | 99.71% | 0.87 |
|  | 8 | 1332/57920 and 148/6480 | 94.59% | 99.87% | 99.75% | 0.89 |
|  | 9 | 1332/57920 and 148/6380 | 89.86% | 99.78% | 99.55% | 0.81 |
|  | 10 | 1332/57920 and 148/6380 | 90% | 99.78% | 99.56% | 0.81 |
|  | **AVE** |  | 92.11% | 99.79% | 99.61% | 83.50% |
|  | **S.D** |  | 0.01540 | 0.00090 | 0.00088 | 0.03136 |
|  | **S.E.M** |  | 0.00487 | 0.00028 | 0.00028 | 0.00992 |
