# Supplementary material for: A Two-Step Target Binding and Selectivity Support Vector Machines Approach for Virtual Screening of Dopamine Receptor Subtype-Selective Ligands
Source: PLoS One. 2012 Jun 15;7(6):e39076. doi: 10.1371/journal.pone.0039076 (PMC3376116; doi:10.1371/journal.pone.0039076)
Supplement: Table S3 — List of 98 molecular descriptors computed by using our own developed MODEL program. (DOC) [file pone.0039076.s007.doc]

**Supplementary Table S3** List of 98 molecular descriptors computed by using our own developed MODEL program.

| **Descriptor Class** | **No of Descriptors in Class** | **Descriptors** |
| --- | --- | --- |
| Simple molecular properties | 18 | Number of C,N,O,P,S, Number of total atoms, Number of rings, Number of bonds, Number of non-H bonds, Molecular weight,, Number of rotatable bonds, number of H-bond donors, number of H-bond acceptors, Number of 5-member aromatic rings, Number of 6-member aromatic rings, Number of N heterocyclic rings, Number of O heterocyclic rings, Number of S heterocyclic rings. |
| Chemical properties | 3 | Sanderson electronegativity, Molecular polarizability, aLogp |
| Molecular Connectivity and shape | 35 | Schultz molecular topological index, Gutman molecular topological index, Wiener index, Harary index, Gravitational topological index, Molecular path count of length 1-6, Total path count, Balaban Index J, 0-2th valence connectivity index, 0-2th order delta chi index, Pogliani index, 0-2th Solvation connectivity index, 1-3th order Kier shape index, 1-3th order Kappa alpha shape index, Kier Molecular Flexibility Index, Topological radius, Graph-theoretical shape coefficient, Eccentricity, Centralization, Logp from connectivity. |
| Electro-topological state | 42 | Sum of Estate of atom type sCH3, dCH2, ssCH2, dsCH, aaCH, sssCH, dssC, aasC, aaaC, sssC, sNH3, sNH2, ssNH2, dNH, ssNH, aaNH, dsN, aaN, sssN, ddsN, aOH, sOH, ssO, sSH; Sum of Estate of all heavy atoms, all C atoms, all hetero atoms, Sum of Estate of H-bond acceptors, Sum of H Estate of atom type HsOH, HdNH, HsSH, HsNH2, HssNH, HaaNH, HtCH, HdCH2, HdsCH, HaaCH, HCsats, HCsatu, Havin, Sum of H Estate of H-bond donors |
