# Supplementary material for: A Two-Step Target Binding and Selectivity Support Vector Machines Approach for Virtual Screening of Dopamine Receptor Subtype-Selective Ligands
Source: PLoS One. 2012 Jun 15;7(6):e39076. doi: 10.1371/journal.pone.0039076 (PMC3376116; doi:10.1371/journal.pone.0039076)
Supplement: Table S4 — Results of 5-fold cross validation (CV) tests of SVM models in predicting D1, D2, D3 and D4 ligands. SE, SP, Q and C are sensitivity, specificity, overall accuracy and Matthews correlation coefficient respectively. (DOC) [file pone.0039076.s008.doc]

**Supplementary Table S4** Results of 5-fold cross validation (CV) tests of SVM models in predicting D1, D2, D3 and D4 ligands. SE, SP, Q and C are sensitivity, specificity, overall accuracy and Matthews correlation coefficient respectively.

| **Dopamine Receptor Subtype** | **5-fold CV Tests for Parameter Selection Based on the Training Datasets in Table 1** | | | | | | **5-fold CV Tests for Performance Evaluation Based on the Independent Testing Datasets in Table 1** | | | |
| --- | --- | --- | --- | --- | --- | --- | --- | --- | --- | --- |
| D1 | **Fold** | **Number of ligands/non-ligands** | **SE** | **SP** | **Q** | **C** | **Number of ligands/non-ligands** | **SE** | **SP** | **Q** |
|  | 1 | 99/13092 | 91.92% | 99.87% | 99.81% | 0.77 | 59/25 | 71.19% | 76.00% | 72.62% |
|  | 2 | 99/13093 | 88.78% | 99.91% | 99.83% | 0.78 | 59/25 | 72.88% | 72.00% | 71.43% |
|  | 3 | 98/13093 | 88.78% | 99.87% | 99.79% | 0.74 | 59/25 | 71.19% | 72.00% | 71.43% |
|  | 4 | 98/13092 | 87.76% | 99.88% | 99.79% | 0.74 | 59/25 | 71.19% | 72.00% | 71.43% |
|  | 5 | 97/13093 | 87.76% | 99.92% | 99.83% | 0.78 | 59/25 | 71.19% | 72.00% | 71.43% |
|  | **AVE** |  | 89.00% | 99.89% | 99.81% | 0.76 |  | 71.53% | 72.80% | 71.67% |
|  | **S.D** |  | 0.01710 | 0.00024 | 0.0002 | 0.02049 |  | 0.00756 | 0.01789 | 0.00532 |
|  | **S.E.M** |  | 0.00765 | 0.00011 | 0.00009 | 0.00917 |  | 0.00338 | 0.008 | 0.00238 |
| D2 | **Fold** | **Number of ligands/non-ligands** | **SE** | **SP** | **Q** | **C** | **Number of ligands/non-ligands** | **SE** | **SP** | **Q** |
|  | 1 | 441/13092 | 92.74% | 99.66% | 99.44% | 0.83 | 135/65 | 86.67% | 61.54% | 78.50% |
|  | 2 | 441/13092 | 94.10% | 99.70% | 99.52% | 0.86 | 135/65 | 88.15% | 63.08% | 80.00% |
|  | 3 | 440/13093 | 93.12% | 99.68% | 99.47% | 0.84 | 135/65 | 86.67% | 63.08% | 79.00% |
|  | 4 | 440/13093 | 91.82% | 99.67% | 99.42% | 0.82 | 135/65 | 85.93% | 67.69% | 80.00% |
|  | 5 | 440/13092 | 91.82% | 99.58% | 99.33% | 0.80 | 135/65 | 85.93% | 61.54% | 78.00% |
|  | **AVE** |  | 92.72% | 99.66% | 99.44% | 0.83 |  | 86.67% | 63.39% | 79.10% |
|  | **S.D** |  | 0.00960 | 0.00046 | 0.00070 | 0.02236 |  | 0.00906 | 0.02526 | 0.00894 |
|  | **S.E.M** |  | 0.00429 | 0.00021 | 0.00031 | 0.01 |  | 0.00405 | 0.01130 | 0.004 |
| D3 | **Fold** | **Number of ligands/non-ligands** | **SE** | **SP** | **Q** | **C** | **Number of ligands/non-ligands** | **SE** | **SP** | **Q** |
|  | 1 | 271/12712 | 90.77% | 99.75% | 99.56% | 0.80 | 76/28 | 86.84% | 64.29% | 79.81% |
|  | 2 | 271/12712 | 93.73% | 99.79% | 99.66% | 0.84 | 76/28 | 89.47% | 67.86% | 82.69% |
|  | 3 | 271/12712 | 92.99% | 99.77% | 99.63% | 0.83 | 76/28 | 86.84% | 64.29% | 79.81% |
|  | 4 | 271/12711 | 89.67% | 99.82% | 99.61% | 0.82 | 76/28 | 84.21% | 67.86% | 79.81% |
|  | 5 | 271/12711 | 95.20% | 99.88% | 99.78% | 0.90 | 76/28 | 89.47% | 64.29% | 81.73% |
|  | **AVE** |  | 92.47% | 99.80% | 99.65% | 0.84 |  | 87.37% | 65.72% | 80.77% |
|  | **S.D** |  | 0.02238 | 0.00051 | 0.00082 | 0.03768 |  | 0.022 | 0.01955 | 0.0136 |
|  | **S.E.M** |  | 0.01001 | 0.00023 | 0.00037 | 0.01685 |  | 0.00984 | 0.00875 | 0.00608 |
| D4 | **Fold** | **Number of ligands/non-ligands** | **SE** | **SP** | **Q** | **C** | **Number of ligands/non-ligands** | **SE** | **SP** | **Q** |
|  | 1 | 297/12760 | 95.29% | 99.71% | 99.61% | 0.84 | 29/33 | 89.66% | 72.73% | 80.65% |
|  | 2 | 297/12760 | 93.27% | 99.76% | 99.61% | 0.84 | 29/33 | 86.21% | 63.64% | 74.19% |
|  | 3 | 297/12760 | 94.28% | 99.80% | 99.67% | 0.86 | 29/33 | 89.66% | 63.64% | 75.81% |
|  | 4 | 297/12759 | 93.94% | 99.79% | 99.66% | 0.85 | 29/33 | 86.21% | 63.64% | 74.19% |
|  | 5 | 297/12759 | 94.61% | 99.76% | 99.65% | 0.85 | 29/33 | 86.21% | 63.64% | 74.19% |
|  | **AVE** |  | 94.28% | 99.76% | 99.64% | 0.848 |  | 87.59% | 65.46% | 75.81% |
|  | **S.D** |  | 0.00752 | 0.00035 | 0.00028 | 0.00837 |  | 0.0189 | 0.04065 | 0.02794 |
|  | **S.E.M** |  | 0.00337 | 0.00015 | 0.00013 | 0.00374 |  | 0.00845 | 0.01818 | 0.01249 |
