# Supplementary material for: A Two-Step Target Binding and Selectivity Support Vector Machines Approach for Virtual Screening of Dopamine Receptor Subtype-Selective Ligands
Source: PLoS One. 2012 Jun 15;7(6):e39076. doi: 10.1371/journal.pone.0039076 (PMC3376116; doi:10.1371/journal.pone.0039076)
Supplement: Table S5 — Numbers of Pubchem compounds at different similarity levels with respect to known ligands of each dopamine receptor subtype, and percent of these compounds identified by SVM VS model as subtype selective ligands. (DOC) [file pone.0039076.s009.doc]

**Supplementary Table S5** Numbers of Pubchem compounds at different similarity levels with respect to known ligands of each dopamine receptor subtype, and percent of these compounds identified by SVM VS model as subtype selective ligands.

| Dopamine receptor subtype |  | Similarity level with respect to known ligands of the subtype defined by Tanimoto similarity score | | | | | | | | | |
| --- | --- | --- | --- | --- | --- | --- | --- | --- | --- | --- | --- |
|  |  | 0-0.1 | 0.1-0.2 | 0.2-0.3 | 0.3-0.4 | 0.4-0.5 | 0.5-0.6 | 0.6-0.7 | 0.7-0.8 | 0.8-0.9 | 0.9-1 |
| D1 | Number of Pubchem compounds at the similarity level | 366852 | 1238930 | 2210766 | 3832638 | 3652430 | 781974 | 384551 | 355589 | 339499 | 389378 |
|  | Percent of these Pubchem compounds identified as subtype selective ligand | 0.0499% | 0.0489% | 0.0498% | 0.0521% | 0.0509% | 0.0493% | 0.0486% | 0.0515% | 0.0510% | 0.0507% |
| D2 | Number of Pubchem compounds at the similarity level | 477873 | 1111819 | 1464190 | 1707149 | 3026529 | 2593708 | 892995 | 659690 | 812545 | 806109 |
|  | Percent of these Pubchem compounds identified as subtype selective ligand | 0.1306% | 0.1320% | 0.1322% | 0.1350% | 0.1311% | 0.1306% | 0.1303% | 0.1326% | 0.1309% | 0.1310% |
| D3 | Number of Pubchem compounds at the similarity level | 770711 | 1497979 | 2325005 | 3232481 | 1718412 | 896213 | 664517 | 662908 | 812545 | 650036 |
|  | Percent of these Pubchem compounds identified as subtype selective ligand | 0.1445% | 0.1471% | 0.1434% | 0.1475% | 0.1467% | 0.1456% | 0.1477% | 0.1469% | 0.1470% | 0.1473% |
| D4 | Number of Pubchem compounds at the similarity level | 947701 | 1348342 | 2672549 | 2548656 | 2350749 | 942874 | 778756 | 662908 | 733704 | 566368 |
|  | Percent of these Pubchem compounds identified as subtype selective ligand | 0.1601% | 0.1593% | 0.1579% | 0.1568% | 0.1580% | 0.1591% | 0.1588% | 0.1576% | 0.1582% | 0.1579% |
