# Supplementary material for: A Two-Step Target Binding and Selectivity Support Vector Machines Approach for Virtual Screening of Dopamine Receptor Subtype-Selective Ligands
Source: PLoS One. 2012 Jun 15;7(6):e39076. doi: 10.1371/journal.pone.0039076 (PMC3376116; doi:10.1371/journal.pone.0039076)
Supplement: Table S6 — The performance of our new method 2SBR-SVM and that of previously used methods Combi-SVM, ML-kNN and RAkEL-DT in predicting estrogen receptor subtype selective and multi-subtype ligands. (DOC) [file pone.0039076.s010.doc]

**Supplementary Table S6** The performance of our new method 2SBR-SVM and that of previously used methods Combi-SVM, ML-kNN and RAkEL-DT in predicting estrogen receptor subtype selective and multi-subtype ligands.

| **Type of estrogen receptor ligands** | **Number of ligands** | **Percent of these ligands correctly identified by method** | | | |
| --- | --- | --- | --- | --- | --- |
|  |  | Combi-SVM | ML-kNN | RAkEL-DT | 2SBR-SVM |
| ERα selective ligands | 40 | 55.00% | 40.00% | 52.50% | 85.00% |
| ERβ selective ligands | 55 | 60.00% | 54.55% | 58.18% | 80.00% |
| ERα and ERβ multi-subtype ligands | 63 | 63.49% | 44.44% | 49.20% | 69.84% |
