# Supplementary material for: A Two-Step Target Binding and Selectivity Support Vector Machines Approach for Virtual Screening of Dopamine Receptor Subtype-Selective Ligands
Source: PLoS One. 2012 Jun 15;7(6):e39076. doi: 10.1371/journal.pone.0039076 (PMC3376116; doi:10.1371/journal.pone.0039076)
Supplement: Table S7 — Virtual screening performance of our new method 2SBR-SVM and that of our previously used method Combi-SVM in scanning 13.56 million Pubchem compounds, 168,016 MDDR compounds and 657,736 ChEMBLdb compounds. For comparison, the results of single label SVM, which identify putative subtype binding ligands regardless of their possible binding to another subtypes, are also included. (DOC) [file pone.0039076.s011.doc]

**Supplementary Table S7** Virtual screening performance of our new method 2SBR-SVM and that of our previously used method Combi-SVM in scanning 13.56 million Pubchem compounds, 168,016 MDDR compounds and 657,736 ChEMBLdb compounds. For comparison, the results of single label SVM, which identify putative subtype binding ligands regardless of their possible binding to another subtypes, are also included.

| Estrogen receptor subtype | Method | Number and Percent of the 13.56M PubChem Compounds Identified as subtype selective ligands | Number and Percent of the 168,016 MDDR Compounds Identified as subtype selective ligands | Number and Percent of the 657,736 ChemBL Compounds Identified as subtype selective ligands |
| --- | --- | --- | --- | --- |
| ERalpha | SVM (Single Label) | 19508(0.1439%) | 1395(0.8303%) | 2689(0.4088%) |
|  | Combi-SVM | 9570(0.0706%) | 1075(0.6398%) | 1931(0.2936%) |
|  | 2SBR-SVM | 1279(0.0094%) | 107(0.0637%) | 221(0.0336%) |
| ERbeta | SVM (Single Label) | 20067(0.1480%) | 1167(0.6946%) | 3017(0.4587%) |
|  | Combi-SVM | 10756(0.0793%) | 768(0.4571%) | 1562(0.2375%) |
|  | 2SBR-SVM | 1364(0.0101%) | 94(0.0559%) | 215(0.0327%) |
